# Efficient One-Pot Synthesis of Novel Caffeic Acid Derivatives as Potential Antimalarials

**DOI:** 10.1155/2023/6675081

**Published:** 2023-11-24

**Authors:** Katarzyna Sidoryk, Silvia Parapini, Nicoletta Basilico, Magdalena Zaremba-Czogalla, Marek Kubiszewski, Marcin Cybulski, Jerzy Gubernator, Agnieszka Zagórska, Anna Jaromin

**Affiliations:** ^1^Pharmacy, Cosmetic Chemistry and Biotechnology Research Group, Łukasiewicz Research Network-Industrial Chemistry Institute, Warsaw, Poland; ^2^Dipartimento di Scienze Biomediche per la Salute, Università di Milano, Milan, Italy; ^3^Dipartimento di Scienze Biomediche, Chirurgiche e Odontoiatriche, Università di Milano, Milan, Italy; ^4^Department of Lipids and Liposomes, Faculty of Biotechnology, University of Wroclaw, Wroclaw, Poland; ^5^Pharmaceutical Analysis Laboratory, Łukasiewicz Research Network-Industrial Chemistry Institute, Warsaw, Poland; ^6^Department of Medicinal Chemistry, Jagiellonian University Medical College, Cracow, Poland

## Abstract

New protocol for the preparation of the novel caffeic acid derivatives using the Wittig reaction has been applied to follow the principles of green chemistry. The compounds have been evaluated against chloroquine-sensitive and chloroquine-resistant *P. falciparum* strains. Their cytotoxicity to normal human dermal fibroblasts and their propensity to induce hemolysis have been also determined. Ethyl (2*E*)-3-(2,3,4-trihydroxyphenyl)-2-methylpropenoate has exhibited the highest antiplasmodial activity against *P. falciparum* strains without the cytotoxic and hemolytic effects. This derivative is significantly more potent than caffeic acid parent structure. The application of our one-step procedure has been shown to be rapid and efficient. It allows for an easy increase of input data to refine the structure-activity relationship model of caffeates as the antimalarials. The one-step approach meets the conditions of “atom economy” and eliminates hazardous materials. Water has been used as the effective medium for the Wittig reaction to avoid toxic organic solvents.

## 1. Introduction

**Figure 1:**
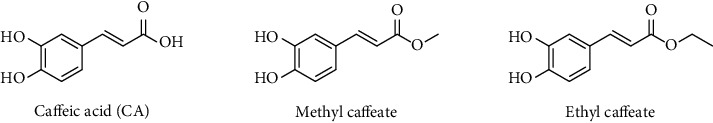
Structures of caffeic acid (CA) and its derivatives from [15] with antimalarial activity.

Malaria is an important public health problem in tropical and subtropical countries. The World Health Organization (WHO) reported that there were an estimated 247 million malaria cases in 2021 which was alarming increase from 245 million in 2020 [1]. Malaria is caused by protozoan parasites of the genus *Plasmodium*. To date, over two hundred species of *Plasmodium* have been formally described, and each species infects a certain range of hosts. *Plasmodium* species that cause malaria in humans in the large areas of the world are limited to five species: *P. falciparum*, *P. vivax*, *P. malariae*, *P. ovale*, and *P. knowlesi* [2]. First-line malaria treatment relies on artemisinin-based combination therapies (ACTs) in association with partner drugs, such as lumefantrine, amodiaquine, mefloquine, or piperaquine [1, 3]. These therapeutic options are increasingly threatened by parasite mutation and the occurrence of drug-resistant parasite strains. Recently, the evidence of partial artemisinin resistance against *P. falciparum*, with mutations in the Pfkelch13 and Pfmdr1 multidrug resistance-1 genes, has been observed in Rwandan patients. Nevertheless, artemether-lumefantrine therapy remains effective but with reduced efficacy [4, 5]. Positively, malaria vaccine RTS, S/AS01 (RTS, S) recommended by the WHO and other malaria vaccine candidates may change the situation [1]. Despite this, new single-use antimalarial drugs with broad therapeutic potential and new mechanisms of action are still needed to make it more difficult for parasites to act [6]. Research on new antimalarial agents may be gathered into two approaches: the search for new compounds of natural or synthetic origin and the direct development of phytomedicines. Extracts from South African plant species containing a phenolic fraction have been identified as traditional therapies for malaria. In the studies, these extracts were effective against the parasite during the hepatic stage [7–10]. Caffeic acid (CA) ((*E*)-3-(3,4-dihydroxyphenyl)prop-2-enoic acid) (Figure 1) is an orally bioavailable polyphenol acid. CA is synthesized by plants as a secondary metabolite. This compound and its derivatives exhibit antibacterial [11], antiviral [12], anticancer [13], and anti-inflammatory properties [14]. Moreover, CA has been the most potent of the phenolic acids tested for antimalarial activities [15, 16]. Caffeic acid and seven synthetic derivatives were evaluated for their in vitro antimalarial activity against *P. falciparum*, with artemisinin-positive control, to recognize the methyl and ethyl esters of caffeic acid (Figure 1) as the most active *in vitro*. Thus, both compounds were then evaluated for antimalarial activity *in vivo*. Ethyl caffeate showed 55% at 100 mg/kg of *P. berghei* inhibition growth, acting by blocking young parasitic forms [16].

Most common commercially available polyphenolic acids are usually obtained by extraction from their natural plant sources, as well as in the biotechnological process [17]. The chemical synthesis also plays a significant role in accessing pure polyphenolic acids. The CA in its structure possess the *α*,*β*-unsaturated carbonyl units; thus, to obtain this substructure part, the Wittig reaction, the Horner-Wadsworth-Emmons modification [18] or Knoevenagel [19], and aldol condensations [20] are commonly used strategies. The HWE modification introduces the use of nontoxic dialkyl-2-oxopropylphosphonates in reaction involved in *α*,*β*-unsaturated compound formation. Moreover, the HWE reaction preferentially gives stable *E* isomers of *α*,*β*-unsaturated esters and ketones, which are employed in pharmaceutical synthesis. On the other hand, studies on the HWE revealed some limitations such as the use of toxic solvents, strong bases or expensive catalysts, and moderate yields [21]. Several variations in the Wittig and HWE reaction conditions have been reported, and one of them demonstrated that the water medium can be very effective [22]. Recently, we have developed a high-yield one-step procedure for synthesizing derivatives of caffeic acid with variable numbers of hydroxyl groups in the aromatic ring and with methyl ester or acetyl terminal group [23].

To better understand the impact of structural modifications in the aromatic ring of caffeates on antiplasmodial activity, there was a need to increase the number of new chemical entities derived from CA. In this study, we investigated the possible antimalarial activity of new double- and three-point modified CA analogs. The modifications included the ethyl or *tert*-butyl esters with different substituent at hydroxyl positions or additional hydroxyl groups. Ester derivatives with a fluorine atom introduced into the aromatic ring and with an additional methyl group at the double bond were also synthesized and biologically evaluated. A high-yield, one-step procedure for the synthesis of CA derivatives was adopted to synthesize the new compounds [23]. The compounds were analyzed for antiplasmodial activity and toxicity against NHDF cells, and their potential as antimalarial compounds was discussed.

## 2. Results and Discussion

### 2.1. Chemistry

The new derivatives were obtained according to the method that relies on the Wittig reaction. Thus, the commercially available aldehydes with a fluor atom substituent and/or additional hydroxyl groups were used as starting materials for a one-step reaction with appropriate ylides. The structures of reaction products **1-6** were confirmed by ^1^H and ^13^C NMR analysis (Supplementary Materials), as well as HRMS experiments. In all examples, the Wittig conditions gave the products with good yields (48-94%, Table 1). For compounds **1**, **2**, **4**, and **6**, vicinal coupling data ranged from 15.9 to 16.1 Hz, which confirmed their structure as *E* isomers. For compounds **3** and **5**, the vicinal coupling was not observed because of the presence of only single proton near double bond. However, all ylides used by us are classified as stabilized one, which give in theory and practice *E* products due to dipole-dipole interactions [24].

Despite their simple structures, compounds **1-5** have been unknown, or the published protocols for their synthesis have been unsatisfactory, as for compound **6** [25]. Despite the unquestionable advantages of reaction in water, due to its stabilizing solvation effects on ylide substrates, there are no literature reports on its suitability for aromatic aldehydes containing more than one unprotected hydroxyl group. Moreover, the disclosed methods for the synthesis of the caffeic acid derivatives do not meet the principles of green chemistry, e.g., “atom economy,” safer solvents, and catalysts. They are also not beneficial, neither because of harsh reaction conditions nor the low yield. In detail, compound **1** (*tert*-butyl (*E*)-3-(2,4-dihydroxyphenyl)cinnamate) can be synthetized after the cleavage of the lactone bond of 7-hydroxycoumarin in the presence of potassium *tert*-butoxide (*tert*-BuOK) in dimethylformamide (DMF) (80°C), under nitrogen atmosphere [26]. The tri-OH-substituted CA derivatives were previously obtained by the Knoevenagel condensation between an appropriate 3,4,5-trihydroxybenzaldehyde precursor and diethylmalonate in pyridine, in the presence of piperidine [27] or aniline [28, 29] catalysts. However, this procedure failed when 2,3,4-trihydroxybenzaldehyde substrate was applied. Compound with three hydroxyl groups at the 3,4,5 position of phenyl ring was described by the Ducrot group [30]. The three-step procedure consisted of hydroxyl group peracetylation, Horner-Wadsworth-Emmons-Wittig olefination, and acetate removal to give product with an overall yield of 53%. Although the synthesis of **6** through the Heck reaction was discussed previously [25], no experimental data has been revealed, except for the reaction yield of 52%.

To sum up, our protocol for the preparation of the CA derivatives using the Wittig reaction met the principles of green chemistry [31]. Firstly, water was used as the effective reaction medium for aromatic aldehydes as the substrates with more than one unprotected hydroxyl group. The protocol replaced toxic organic solvents such as DMF and one-step reaction applied “atom economy.” The other reaction materials such as additional solvents and separating agents were also eliminated. Moreover, environmentally unsafe reagents (e.g., BBr_3_) and harsh reaction conditions were excluded. Finally, the application of our one-step procedure for different CA derivatives turned to be rapid and effective, while compared to existing procedures, our protocol gave compounds **2**, **3**, and **4** in a short reaction time with moderate yields of 52%, 74%, and 48%, respectively. Related results were observed for the synthesis of compounds **5** and **6** with 3-fluoro-4-hydroxybenzaldehyde and corresponding ylides as the substrates. For compound **6**, the 74% reaction yield was higher when compared to 52-53% from literature [25, 30]. The results for aldehydes with three hydroxyls in the phenyl ring revealed the electron donating effect (EDG) as the most influencing on partial positive charge on the carbonyl group and susceptibility to aldehyde reactivity with ylides.

### 2.2. Biological Studies

Analysis of physicochemical parameters of compounds **1-6** revealed that the most potent compounds **2-4** exhibited the lowest values of log *P*_o/w_ in the range of 1.34-1.92 (Table 2). On the other hand, the transport of compounds into cells and across membranes is a function of physicochemical properties and the acid-base property of a molecule (expressed as pK_a_). The number of hydroxyl groups in the structure determines the possibility of ionization of studied compounds. Compounds **5** and **6** had a single pK_a_ value, related to one hydroxyl group, compound **1** exhibited two pK_a_ states, whereas compounds **2**-**4** had three (Table 2). Based on the pK_a_ values, it can be concluded that they all underwent slight ionization in the physiological pH range, which reduces their solubility in a polar environment and diffusion across the membranes. However, the lipophilic properties of chloroquine (CQ) indicate optimal membrane transfer at pH 7.4, and the drug can diffuse across the *P. falciparum* food vacuole (*Pf* FV). Next, in the acidic environment of the FV, CQ is protonated and is subsequently unable to freely diffuse out of the FV again. Thus, the log *D* values were established at pH 7.4 (blood), 7.2 (cytoplasm), and 5.5 (*Pf* FV) to explore an accumulation of compounds in the *Pf* FV, which could impact their antimalarial activity (Table 2). A comparison between log *P*_o/w_ and log *D* 7.4 revealed an increase in lipophilicity of one log unit. However, neither compound exhibited log *D* parameters that would favor accumulation in the FV of the parasite.

According to our literature survey, there are only few reports describing antimalarial activity of caffeic acid and its derivatives [16, 32]. Due to the shortage of SAR data on the antiplasmodial activity of this type of natural derivatives, we determined the antiplasmodial activities of our new compounds against chloroquine-sensitive (D10) and chloroquine-resistant (W2) *P. falciparum* strains. Caffeic acid (CA) and chloroquine (CQ), a known antimalarial drug, were chosen as the reference compounds.

As shown in Table 3, the least active compounds were **5** and **6**, with IC_50_ values of 117.42 and 145.08 *μ*M, respectively, which were the ester analogs with a fluorine atom introduced to the aromatic ring and endowed with only one hydroxyl group. Compounds **1** and **2** were slightly more active, while the most active were compounds **3** and **4**, showing an IC_50_ in a very narrow range from 10.96 to 12.71 *μ*M, respectively. It is worth mentioning that these IC_50_ values were better than those obtained by Degotte et al. [32] for other caffeic acid derivatives with values greater than 16 *μ*M. Interestingly, from the compounds **2**, **3**, and **4** each having three -OH groups in the phenyl ring, only the last two exhibited the highest parasite inhibitory activity. The role of the hydroxyl substituent was especially evident when comparing the activities of **1** and **4**. The introduction of an additional hydroxyl group in **4** resulted in a significant decrease of the IC_50_ value, which was even 7.9 times less in the case of a CQ-resistant strain. Another interesting observation was that all the newly synthesized compounds showed significantly better activity against *Plasmodium* strains than their parent compound (caffeic acid), with IC_50_ values greater than 277.53 *μ*M. Our findings correlated well with those of Alson et al., who reported that phenolic acid ester derivatives exhibit better antiplasmodial activity than the corresponding caffeic or chlorogenic acids [16]. Also, all compounds, except **1**, were more effective against the CQ resistant strain, as reflected by the low resistance indexes (RI). Such an improvement in the activity of our novel caffeic acid derivatives was particularly desirable since *P. falciparum* had developed resistance to existing antimalarial agents [34, 35]. Compounds **2**-**6** had RI values ≤ 0.92, suggesting no cross-resistance with chloroquine and the possibility to be considered as promising agents against both the sensitive and resistant strains of the *Plasmodium* parasite.

In parallel, the novel molecules were also screened against a normal human dermal fibroblast cell line, to assess the cytotoxicity of these compounds and estimate their selectivity. To be considered as antiplasmodial, the compounds should exhibit satisfactory selectivity towards *Plasmodium* parasites, but with minimal cytotoxicity directed towards host mammalian cells. As demonstrated in Table 3, compounds **1**, **3**, and **4** showed the highest selectivity, expressed as SI (selectivity index) values (6.23-8.73). However, **1** was less selective in the case of the CQ-resistant strain, with an SI of 2.89. Additionally, to better study the cytotoxicity of the most active and selective agents, namely, **3** and **4**, their hemolytic activity against human erythrocytes was studied. This aspect is especially important as publications report the occurrence of hemolysis after administration of antimalarial drugs [36, 37] or even after contact with other bioactive acids [38]. We investigated the possible cytotoxicity of the tested compounds at the concentration of 12.5 *μ*M, which corresponded to IC_50_ for both strains of *Plasmodium*. At this concentration, **3** showed only 4.13 ± 0.85% hemolysis, while incubation with compound 4 resulted in a high 52.93 ± 2.93% hemolysis. The difference in the results may reflect differences in chemical structures of these compounds, as the crucial factor for biological activity. The only structural difference for **3** was the presence of additional methyl group at double bound. Summarizing the results, compound **4** was not hemocompatible, unlike **3**. Thus, it can be estimated that the high Plasmodium activity of compound **3** will not have a toxic effect on host cells.

## 3. Conclusions

In summary, the novel series of caffeic acid derivatives described in this study were prepared through a simple and environmentally friendly synthesis method. This green one-pot synthesis allows a fast and easy route to obtain various derivatives of CA which can be used both for further chemical modification and biological screening. Interestingly, the antiplasmodial assays revealed that even minor changes in the structure of the CA chemical backbone significantly increase its antiplasmodial activity. Overall, the ester compound **3** with a methyl group located at the double bond position was the most promising compound. We believe that our initial and promising research will bring closer to identifying the most effective antimalarial agent.

## 4. Experimental

### 4.1. Chemistry

#### 4.1.1. General Remarks

All reagents and solvents were purchased from common commercial suppliers without further purification. Merck (Merck, Darmstadt, Germany) DC-Alufolien Kieselgel 60 F254 TLC plates were used for monitoring the progress of the reactions. Column chromatography was performed on Merck (Merck, Darmstadt, Germany) silica gel 60 (230-400 mesh). Melting points were measured on a Mettler-Toledo MP90 (Mettler-Toledo, Columbus, OH, USA) apparatus and were uncorrected. NMR spectra were recorded on a Varian VNMRS 500 (Varian Inc., Palo Alto, CA, USA) spectrometer (at 298 K) in CDCl_3_ and/or CD_3_OD, using TMS as an internal standard. High-resolution mass spectrometry (HRMS) measurements were performed using a Synapt G2-Si mass spectrometer (Waters Corporation, Milford, MA, USA) equipped with an ESI source and quadrupole-time-of-flight mass analyzer. The measurements were performed with the capillary voltage set to 2.7 kV and the sampling cone set to 20 V. The source temperature was 110°C. To ensure accurate mass measurements, data were collected in centroid mode, and mass was corrected during acquisition using leucine encephalin solution as an external reference. The results of the measurements were processed using MassLynx 4.1 software (Waters Corporation, Milford, MA, USA).

#### 4.1.2. General Procedure for the Synthesis of the Wittig Reaction of Compounds 1-6

A suspension of an appropriate aromatic aldehyde (1 eq.) and ylide (1.3-1.5 eq.) in water (4-10 mL) was stirred at 90°C for 0.5-4 h. Next, the heterogeneous reaction mixture was cooled to room temperature, and the aqueous phase was extracted with dichloromethane (3 × 10 mL). The solvent was evaporated under diminished pressure. Column chromatography (hexane-ethyl acetate or chloroform-methanol) of the residue gave the (*E*)-alkene as the major product.

*(1) tert-Butyl (2E)-3-(2,4-Dihydroxyphenyl)-propenoate*. From 2,4-dihydroxybenzaldehyde (0.72 mmol, 100 mg) and (*tert*-butoxycarbonylmethylene)triphenylphosphorane (0.94 mmol, 354 mg). Yield 94%, oil, ^1^H NMR (500 MHz, CDCl_3_+CH_3_OD) *δ* 7.93 (d, 1H, *J* = 16.1 Hz, -CH=CH-), 7.33 (d, 1H, *J* = 8.5 Hz), 6.47 (d, 1H, *J* = 2.3 Hz), 6.42 (dd, 1H, *J* = 8.5 Hz, *J* = 2.3 Hz), 6.37 (d, 1H, *J* = 16.1 Hz, -CH=CH-), 1.53 (s, 9H); ^13^C NMR (125 MHz, CDCl_3_+CH_3_OD) *δ* 168.7, 159.0, 156.6, 139.5, 130.1, 116.8, 114.7, 108.8, 103.4, 80.9, 28.2 (Supplementary Figure 1); HR-MS (ES^−^) for C_13_H_15_O_4_ (M)^−^ calculated: 235.0970. Found: 235.0967.

*(2) Ethyl (2E)-3-(2,3,4-Trihydroxyphenyl)-propenoate*. From 2,3,4-trihydroxybenzaldehyde (0.65 mmol, 100 mg) and (ethoxycarbonylmethylene)triphenylphosphorane (0.97 mmol, 340 mg). Yield 52%, white solid, m.p. 172-173°C; ^1^H NMR (500 MHz, CDCl_3_+CH_3_OD) *δ* 7.80 (d, 1H, *J* = 16.0 Hz, -CH=CH-), 6.83 (d, 1H, *J* = 8.6 Hz,), 6.34 (m, 1H, *J* = 16.0 Hz, -CH=CH-), 6.31 (d, 1H, *J* = 8.6 Hz), 4.15 (q, 2H, *J* = 7.1 Hz), 1.23 (t, 3H, *J* = 7.1 Hz); ^13^C NMR (125 MHz, CDCl_3_+CH_3_OD) *δ* 168.7, 147.3, 145.9, 140.9, 131.9, 120.4, 114.8, 114.3, 107.6, 60.1, 14.0 (Supplementary Figure 2); HR-MS (ES^−^) for C_11_H_11_O_5_ (M)^−^ calculated: 223.0606. Found: 223.0611.

*(3) Ethyl (2E)-3-(2,3,4-Trihydroxyphenyl)-2-methylpropenoate*. From 2,3,4-trihydroxybenzaldehyde (0.65 mmol, 100 mg) and (carbethoxyethylidene)triphenylphosphorane (0.97 mmol, 352 mg). Yield 74%, white solid, m.p. 140°C; ^1^H NMR (500 MHz, CDCl_3_+CH_3_OD) *δ* 7.72 (s, 1H), 6.68 (d, 1H, *J* = 8.6 Hz), 6.34 (d, 1H, *J* = 8.6 Hz), 4.15 (q, 2H, *J* = 7.1 Hz), 2.05 (d, 3H, *J* = 1.2 Hz), 1.24 (t, 3H, *J* = 7.1 Hz); ^13^C NMR (125 MHz, CDCl_3_) *δ* 169.6, 145.8, 144.5, 134.5, 132.0, 125.9, 121.0, 115.5, 106.9, 60.7, 14.0 (Supplementary Figure 3); HR-MS (ES^−^) for C_12_H_13_O_5_ (M)^−^ calculated: 237.0763. Found: 237.0764.

*(4) tert-Butyl (2E)-3-(2,3,4-Trihydroxyphenyl)-propenoate*. From 2,3,4-trihydroxybenzaldehyde (0.65 mmol, 100 mg) and (*tert*-butoxycarbonylmethylene)triphenylphosphorane (0.97 mmol, 365 mg). Yield 48%,white solid, m.p. 175°C; ^1^H NMR (500 MHz, CDCl_3_+CH_3_OD) *δ* 7.72 (d, 1H, *J* = 16.0 Hz, -CH=CH-), 6.84 (d, 1H, *J* = 8.6 Hz), 6.35 (d, 1H, *J* = 8.6 Hz), 6.30 (d, 1H, *J* = 16.0 Hz, -CH=CH-), 1.45 (s, 9H); ^13^C NMR (125 MHz, CDCl_3_+CH_3_OD) *δ* 168.0, 146.9, 145.6, 139.7, 131.9, 120.3, 116.9, 114.5, 107.7, 80.1, 28.0 (Supplementary Figure 4); HR-MS (ES^−^) for C_13_H_15_O_5_ (M)^−^ calculated: 251.0919. Found: 251.0918.

*(5) Ethyl (2E)-3-(3-Fluoro-4-hydroxyphenyl)-2-methylpropenoate*. From 3-fluoro-4-hydroxybenzaldehyde (0.71 mmol, 100 mg) and (carbethoxyethylidene)triphenylphosphorane (0.92 mmol, 335 mg). Yield 92%, foam; ^1^H NMR (500 MHz, CDCl_3_) *δ* 7.56 (brs, 1H), 7.19 (dd, 1H, *J* = 11.7 Hz, *J* = 1.9 Hz), 7.12-7.10 (m, 1H), 7.01 (dd, 1H, *J*1 = *J*2 = 8.7 Hz), 5.58 (brs, 1H), 4.26 (q, 2H, *J* = 7.1 Hz), 2.12 (d, 3H, *J* = 1.4 Hz) 1.34 (t, 3H, *J* = 7.1 Hz); ^13^C NMR (125 MHz, CDCl_3_) *δ* 168.7, 151.5 (d, C-F, *J* = 237.9 Hz), 143.7 (d, *J* = 14.5 Hz), 137.3 (d, *J* = 1.7 Hz), 129.0 (d, *J* = 6.4 Hz), 127.6, 126.9 (d, *J* = 3.0 Hz), 117.1 (d, *J* = 2.3 Hz), 116.8 (d, *J* = 18.9 Hz), 60.9, 14.2 (d, *J* = 7.0 Hz) (Supplementary Figure 5); HR-MS (ES^−^) for C_12_H_12_FO_3_ (M)^−^ calculated: 223.0770. Found: 223.0769.

*(6) tert-Butyl (2E)-3-(3-Fluoro-4-hydroxyphenyl)-propenoate*. From 3-fluoro-4-hydroxybenzaldehyde (0.71 mmol, 100 mg) and (*tert*-butoxycarbonylmethylene)triphenylphosphorane (0.92 mmol, 347 mg). Yield 74%, white solid, m.p. 88.3°C; ^1^H NMR (500 MHz, CDCl_3_) *δ* 7.48 (d, 1H, *J* = 15.9 Hz, -CH=CH-), 7.26 (dd, 1H, *J* = 19.4 Hz, *J* = 2.0 Hz), 7.19 (d, 1H, *J* = 8.6 Hz), 6.99 (dd, 1H, *J*1 = *J*2 = 8.6 Hz), 6.20 (d, 1H, *J* = 15.9 Hz, -CH=CH-), 5.72 (brs, 1H) 1.53 (s, 9H); ^13^C NMR (125 MHz, CDCl_3_) *δ* 166.4 0, 150.1 (d, *J* = 238.4 Hz, C-F), 145.2 (d, *J* = 14.5 Hz), 142.2 (d, *J* = 2.3 Hz), 128.0 (d, *J* = 6.2 Hz), 125.3 (d, *J* = 3.1 Hz), 119.1, 117.5 (d, *J* = 2.2 Hz), 114.4 (d, *J* = 18.8 Hz), 80.6, 28.1 (Supplementary Figure 6); HR-MS (ES^−^) for C_13_H_14_FO_3_ (M)^−^ calculated: 237.0927. Found: 237.0928.

### 4.2. Biological Studies

#### 4.2.1. Materials in Biological Assays

Alpha-MEM and the normal human dermal fibroblast cell line (NHDF) were purchased from Lonza (Warsaw, Poland). Phosphate-buffered saline (PBS), fetal bovine serum (FBS), L-glutamine for cell culture, and 100× antibiotic-antimycotic were purchased from Cytogen (Zgierz, Poland). MTT (3-(4,5-dimethylthiazol-2-yl)-2,5-diphenyltetrazolium bromide) was from Sigma-Aldrich (Poznan, Poland), and dimethyl sulfoxide (DMSO) was obtained from Archem (Kamieniec Wroclawski, Poland). RPMI 1640, HEPES buffer, and glutamine for *P. falciparum* cultures were obtained from EuroClone (Pero, Italy), whereas AlbuMax was obtained from Invitrogen (Milan, Italy).

#### 4.2.2. *P. falciparum* Cultures and Drug Susceptibility Assay

P. falciparum cultures were prepared and maintained according to the method of Trager and Jensen, with slight modifications [39]. The CQ-susceptible strain, D10, and the CQ-resistant strain, W2, were maintained at 5% hematocrit (human type A-positive red blood cells) in RPMI 1640 medium, with the addition of 1% AlbuMax, 0.01% hypoxanthine, 20 mM HEPES, and 2 mM glutamine. All the cultures were maintained in an incubator at 37°C in a standard gas mixture consisting of 1% O_2_, 5% CO_2_, and 94% N_2_. Compounds were dissolved in either water or DMSO and then diluted with a medium to achieve the required concentrations (final DMSO concentration < 1%, which is nontoxic to the parasite). Serial dilutions of the compounds were prepared in 96-well flat-bottomed microplates. Asynchronous cultures with a parasitemia of 1-1.5% and 1% final hematocrit were aliquoted into the plates and incubated with the compounds for 72 h at 37°C. Parasite growth was determined spectrophotometrically (OD650) by measuring the activity of parasite lactate dehydrogenase (pLDH), according to a modified version of the method of Makler and Hinrichs, in control and compound-treated cultures [40]. The antimalarial activity was expressed as 50% inhibitory concentrations (IC_50_); each IC_50_ value was expressed as the mean and standard deviation of at least three separate experiments performed in duplicate.

#### 4.2.3. NHDF Cell Culture

NHDF cells were grown in alpha-MEM medium supplemented likewise with 10% FBS, L-glutamine, and antibiotic-antimycotic. Cultures were maintained at 37°C, in a humidified Innova CO-180 incubator (New Brunswick Scientific, Edison, NJ, USA) in an atmosphere containing 5% CO_2_.

#### 4.2.4. MTT Assay

The cytotoxic effects of CA derivatives were evaluated as described in literature [41, 42] using the standard MTT assay [43]. Briefly, NHDF cells were seeded in a 96-well plate at a density of 5 × 10^3^ cells per well in an alpha-MEM medium. Cells were grown overnight and subsequently exposed to the different concentrations of tested compounds dissolved in DMSO. An equivalent volume of DMSO was used as a negative control. After 72 h of incubation, media containing the test compounds were removed, the cells were washed with sterile PBS, and 50 *μ*L of MTT solution (0.5 mg/mL stock solution was 10 times diluted in the appropriate culture medium) was added to each well for 3 h at 37°C. Subsequently, the MTT solution was removed, the formazan crystals were solubilized in DMSO, and absorbance was recorded using an Asys UVM 340 multiwell plate reader (Cambridge, UK) at 560 nm with a reference wavelength of 670 nm. The % of cell viability was calculated using the following formula: Cell viability (%) = (AT/AC) × 100, where AT is the absorbance of the treatment well (treated cells) and AC is the absorbance of the control well (not treated cells). GraphPad Prism software for Windows (GraphPad Software, La Jolla, CA, USA) was used to calculate IC_50_ values.

#### 4.2.5. Determination of Hemolytic Activity of Caffeic Acid Derivatives

Hemolytic activity was measured as described by Jaromin et al. [44] Compounds 3 and 4, dissolved in DMSO, were added in a volume corresponding to a final concentration of 12.5 *μ*M and then incubated with freshly isolated human red blood cells in PBS buffer at 37°C for 30 min. After centrifugation, the absorbance was determined at 540 nm, to assess the released hemoglobin. In addition, negative (erythrocytes in PBS buffer), positive (erythrocytes in distilled water), and DMSO controls were also performed. The study protocol was approved by the Bioethics Commission at the Lower Silesian Medical Chamber (1/PNHAB/2018).

#### 4.2.6. Calculation of log *P*_o/w_, pK_a_ and log *D*

The partition coefficient between n-octanol and water (consensus log *P*_o/w_) of studied compounds was calculated using the SwissADME web tool [45]. The acid-base property of a molecule (pK_a_) and the distribution coefficient (log *D*) was calculated using the MarvinSketch software (version 20.12.0, ChemAxon Ltd.).

## Figures and Tables

**Table 1:** Synthetic scheme, reaction conditions, and the molecular structures of compounds **1**-**6**.

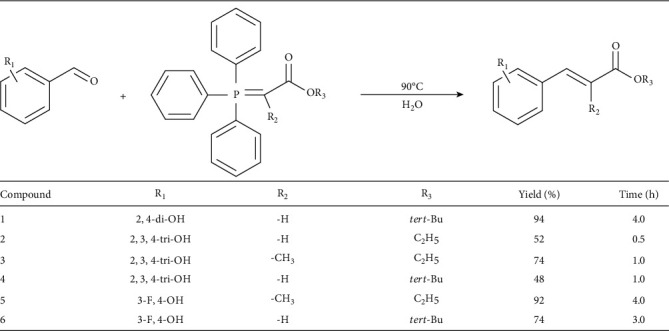


**Table 2:** The log *P*_o/w_, log *D*, and pK_a_ values of compounds **1**-**6**.

Compound	log *P*_o/w_	pK_a_	log *D*		
			pH 7.4	pH 7.2	pH 5.5
1	2.21	9.04 10.40	3.39	3.39	3.40

2	1.34	8.82 10.96 14.49	2.61	2.61	2.62

3	1.61	8.78 10.90 14.46	2.93	2.93	2.94

4	1.92	8.82 10.96 14.49	3.10	3.10	3.11

5	2.63	8.32	3.60	3.62	3.65

6	2.97	8.35	3.78	3.79	3.82

CA	0.93	3.45 9.28 12.69	-1.67	-1.58	-0.21

CQ	4.15	7.29 10.32	0.70	0.44	-1.21

**Table 3:** The antimalarial activity of the series of novel derivatives of caffeic acid against the D10 (CQ-sensitive) and W2 (CQ-resistant) strains of *P. falciparum* and their cytotoxicity to NHDF cells.

Compound	*P. falciparum* IC_50_ (*μ*M)		RI^a^	Cytotoxicity^b^ IC_50_ (*μ*M)	SI_D10_^c^	SI_W2_^d^
	D10	W2				
1	28.63 ± 1.94	86.44 ± 27.45	3.02	249.83 ± 0.51	8.73	2.89
2	42.11 ± 9.12	38.78 ± 17.80	0.92	115.40 ± 1.76	2.74	2.98
3	12.71 ± 2.33	11.29 ± 4.81	0.89	79.22 ± 6.14	6.23	7.02
4	12.18 ± 4.90	10.96 ± 4.88	0.90	90.34 ± 10.32	7.42	8.24
5	145.08 ± 53.63	130.75 ± 52.71	0.90	nd^e^	—	—
6	129.45 ± 59.63	117.42 ± 35.87	0.91	228.75 ± 7.57	1.77	1.95
CA	>277.53	>277.53	—	nd^e^	—	—
CQ	0.02 ± 0.01	0.23 ± 0.08	11.5	40.95 ± 2.29^f^	2047.5	178.04

^a^Ratio between the IC_50_ values of each compound against the W2 and D10 strains of *P. falciparum*. ^b^The cytotoxic activity assayed *in vitro* on NHDF cells using the MTT assay. ^c^SID10 = IC_50_(NHDF)/IC_50_ (D10). ^d^4 SIW2 IC50 (NHDF)/IC_50_ (W2). ^e^Not determined. ^f^Data taken from reference [33].

## Data Availability

The authors confirm that the data supporting the findings of this study are available within the article and its supplementary materials. The other raw data are available from the corresponding authors upon request.

## Supplementary Materials

Supplementary Materials Supplementary Figure 1: *tert*-butyl (2E)-3-(2,4-dihydroxyphenyl)-propenoate (1). Supplementary Figure 2: ethyl (2E)-3-(2,3,4-trihydroxyphenyl)-propenoate (2) 1H and 13C NMR spectra. Supplementary Figure 3: ethyl (2E)-3-(2,3,4-trihydroxyphenyl)-2-methylpropenoate (3) 1H and 13C NMR spectra. Supplementary Figure 4: *tert*-butyl (2E)-3-(2,3,4-trihydroxyphenyl)-propenoate (4) 1H and 13C NMR spectra. Supplementary Figure 5: ethyl (2E)-3-(3-fluoro-4-hydroxyphenyl)-2-methylpropenoate (5) 1H and 13C NMR spectra. Supplementary Figure 6: *tert*-butyl (2E)-3-(3-fluoro-4-hydroxyphenyl)-propenoate (6) 1H and 13C NMR spectra.

## References

[1] World Health Organization (2022). *World malaria report 2022*.

[2] Sato S. (2021). Plasmodium-a brief introduction to the parasites causing human malaria and their basic biology. *Journal of Physiological Anthropology*.

[3] Eastman R. T., Fidock D. A. (2009). Artemisinin-based combination therapies: a vital tool in efforts to eliminate malaria. *Nature Reviews. Microbiology*.

[4] Uwimana A., Umulisa N., Venkatesan M. (2021). Association of *Plasmodium falciparum kelch13* R561H genotypes with delayed parasite clearance in Rwanda: an open-label, single-arm, multicentre, therapeutic efficacy study. *The Lancet Infectious Diseases*.

[5] Elebesunu E. E., Uhuo J. S., Sylvanus P. I. (2021). Antimalarial drug resistance of Plasmodium falciparum in Africa: the need for novel drug treatments. *International Journal of Infectious Diseases*.

[6] Belete T. M. (2020). Recent progress in the development of new antimalarial drugs with novel targets. *Drug Design, Development and Therapy*.

[7] Batista R., Júnior A. D. J. S., De Oliveira A. B. (2009). Plant-derived antimalarial agents: new leads and efficient phytomedicines. Part II. Non-alkaloidal natural products. *Molecules*.

[8] Cock I. E., Selesho M. I., van Vuuren S. F. (2019). A review of the traditional use of southern African medicinal plants for the treatment of malaria. *Ethnopharmacology*.

[9] Builders M. (2019). Antimalarial phenolic compounds in Africa: a review 2019. *European Journal of Biomedical and Pharmaceutical Sciences*.

[10] Ramanandraibe V., Grellier P., Martin M. T. (2008). Antiplasmodial phenolic compounds from Piptadenia pervillei. *Planta Medica*.

[11] Merlani M., Barbakadze V., Amiranashvili L. (2019). New caffeic acid derivatives as antimicrobial agents: design, synthesis, evaluation and docking. *Current Topics in Medicinal Chemistry*.

[12] Langland J., Jacobs B., Wagner C. E., Ruiz G., Cahill T. M. (2018). Antiviral activity of metal chelates of caffeic acid and similar compounds towards herpes simplex, VSV-Ebola pseudotyped and vaccinia viruses. *Antiviral Research*.

[13] Zaremba-Czogalla M., Jaromin A., Sidoryk K., Zagórska A., Cybulski M., Gubernator J. (2020). Evaluation of the in vitro cytotoxic activity of caffeic acid derivatives and liposomal formulation against pancreatic cancer cell lines. *Materials.*.

[14] Shen H., Tong X., Yang J. (2020). Biotransformation of natural hydroxycinnamic acids by gut microbiota from normal and cerebral ischemia-reperfusion injured rats: a comparative study. *Food & Function*.

[15] Andjelković M., Van Camp J., De Meulenaer B. (2006). Iron-chelation properties of phenolic acids bearing catechol and galloyl groups. *Food Chemistry*.

[16] Alson S. G., Jansen O., Cieckiewicz E. (2018). In-vitro and in-vivo antimalarial activity of caffeic acid and some of its derivatives. *The Journal of Pharmacy and Pharmacology*.

[17] Kumar N., Goel N. (2019). Natural versatile molecules with promising therapeutic applications. *Biotechnology Reports*.

[18] Wadsworth W. S. (2005). Synthetic applications of phosphoryl-stabilized anions. *Organic Reactions*.

[19] List B. (2010). Emil Knoevenagel and the roots of aminocatalysis. *Angewandte Chemie (International Ed. in English)*.

[20] Guillena G., Nájera C., Ramón D. J. (2007). Enantioselective direct aldol reaction: the blossoming of modern organocatalysis. *Tetrahedron-Asymmetry*.

[21] Jafari A. A., Ghadami M. (2016). Efficient synthesis of *α*, *β*-unsaturated ketones with trans-selective Horner–Wadsworth–Emmons reaction in water. *Environmental Chemistry Letters*.

[22] El-Batta A., Jiang C., Zhao W., Anness R., Cooksy A. L., Bergdahl M. (2007). Wittig reactions in water media employing stabilized ylides with aldehydes. Synthesis of alpha, beta-unsaturated esters from mixing aldehydes, alpha-bromoesters, and Ph_3_P in aqueous NaHCO_3_. *The Journal of Organic Chemistry*.

[23] Sidoryk K., Jaromin A., Filipczak N., Cmoch P., Cybulski M. (2018). Synthesis and antioxidant activity of caffeic acid derivatives. *Molecules*.

[24] Robiette R., Richardson J., Aggarwal V. K., Harvey J. N. (2006). Reactivity and selectivity in the Wittig reaction: a computational study. *Journal of the American Chemical Society*.

[25] Bernal F. A., Kaiser M., Wünsch B., Schmidt T. J. (2020). Structure-activity relationships of cinnamate ester analogues as potent antiprotozoal agents. *ChemMedChem*.

[26] Punchi Hewage A. N. D., Ya H., Nammalwar B. (2019). Small molecule inhibitors of the BfrB-Bfd interaction decrease Pseudomonas aeruginosa fitness and potentiate fluoroquinolone activity. *Journal of the American Chemical Society*.

[27] Teixeira J., Silva T., Benfeito S. (2013). Exploring nature profits: development of novel and potent lipophilic antioxidants based on galloyl-cinnamic hybrids. *European Journal of Medicinal Chemistry*.

[28] Esteves M., Siquet C., Gaspar A. (2008). Antioxidant versus cytotoxic properties of hydroxycinnamic acid derivatives - a new paradigm in phenolic research. *Archiv der Pharmazie*.

[29] Fiuza S. M., Van Besien E., Milhazes N., Borges F., Marques M. P. M. (2004). Conformational analysis of a trihydroxylated derivative of cinnamic acid--a combined Raman spectroscopy and Ab initio study. *Journal of Molecular Structure*.

[30] Allais F., Martinet S., Ducrot P.-H. (2009). Straightforward total synthesis of 2-O-feruloyl-L-malate, 2-O-sinapoyl-L-malate and 2-O-5-hydroxyferuloyl-L-malate. *Synthesis-Stuttgart*.

[31] Explore the Design Principles of Green & Sustainable Chemistry & Engineering-American Chemical Society. https://www.acs.org/content/acs/en/greenchemistry/principles.html.

[32] Degotte D., Pirotte P., Michel F., Francotte P. (2022). Potential of caffeic acid derivatives as antimalarial leads. *Letters in Drug Design and Discovery*.

[33] Jaromin A., Parapini S., Basilico N. (2021). Azacarbazole n-3 and n-6 polyunsaturated fatty acids ethyl esters nanoemulsion with enhanced efficacy against Plasmodium falciparum. *Bioactive Materials*.

[34] Chinampa M., Via A., Marcatili P., Tramontano A. (2010). On the mechanism of chloroquine resistance in Plasmodium falciparum. *PLoS One*.

[35] Wicht K. J., Mok S., Fidock D. A. (2020). Molecular mechanisms of drug resistance in Plasmodium falciparum malaria. *Annual Review of Microbiology*.

[36] Rehman K., Lötsch F., Kremsner P. G., Ramharter M. (2014). Haemolysis associated with the treatment of malaria with artemisinin derivatives: a systematic review of current evidence. *International Journal of Infectious Diseases*.

[37] Savargaonkar D., Das M. K., Verma A. (2020). Delayed haemolysis after treatment with intravenous artesunate in patients with severe malaria in India. *Malaria Journal*.

[38] Stasiuk M., Jaromin A., Kozubek A. (2004). The effect of merulinic acid on biomembranes. *Biochimica et Biophysica Acta*.

[39] Trager W., Jensen J. B. (1976). Human malaria parasites in continuous culture. *Science*.

[40] Makler M. T., Hinrichs D. J. (1993). Measurement of the lactate dehydrogenase activity of Plasmodium falciparum as an assessment of parasitemia. *The American Journal of Tropical Medicine and Hygiene*.

[41] Fandzloch M., Jaromin A., Zaremba-Czogalla M. (2020). Nanoencapsulation of a ruthenium(ii) complex with triazolopyrimidine in liposomes as a tool for improving its anticancer activity against melanoma cell lines. *Dalton Transactions*.

[42] Filipczak N., Jaromin A., Piwoni A. (2019). A triple co-delivery liposomal carrier that enhances apoptosis via an intrinsic pathway in melanoma cells. *Cancers*.

[43] Mosmann T. (1983). Rapid colorimetric assay for cellular growth and survival: application to proliferation and cytotoxicity assays. *Journal of Immunological Methods*.

[44] Jaromin A., Korycińska M., Piętka-Ottli M. (2012). Membrane perturbations induced by new analogs of neocryptolepine. *Biological & Pharmaceutical Bulletin*.

[45] Daina A., Michielin O., Zoete V. (2017). SwissADME: a free web tool to evaluate pharmacokinetics, drug-likeness and medicinal chemistry friendliness of small molecules. *Scientific Reports*.

