# Supplementary material for: Efficient One-Pot Synthesis of Novel Caffeic Acid Derivatives as Potential Antimalarials
Source: J Parasitol Res. 2023 Nov 24;2023:6675081. doi: 10.1155/2023/6675081 (PMC10691883; doi:10.1155/2023/6675081)
Supplement: Supplementary Materials — Supplementary Figure 1: tert-butyl (2E)-3-(2,4-dihydroxyphenyl)-propenoate (1). Supplementary Figure 2: ethyl (2E)-3-(2,3,4-trihydroxyphenyl)-propenoate (2) 1H and 13C NMR spectra. Supplementary Figure 3: ethyl (2E)-3-(2,3,4-trihydroxyphenyl)-2-methylpropenoate (3) 1H and 13C NMR spectra. Supplementary Figure 4: tert-butyl (2E)-3-(2,3,4-trihydroxyphenyl)-propenoate (4) 1H and 13C NMR spectra. Supplementary Figure 5: ethyl (2E)-3-(3-fluoro-4-hydroxyphenyl)-2-methylpropenoate (5) 1H and 13C NMR spectra. Supplementary Figure 6: tert-butyl (2E)-3-(3-fluoro-4-hydroxyphenyl)-propenoate (6) 1H and 13C NMR spectra. [file 6675081.f1.docx]

**Efficient one-pot synthesis of novel caffeic acid derivatives as a potential antimalarials**

Katarzyna Sidoryk^1^, Silvia Parapini^2^, Nicoletta Basilico^3^, Magdalena Zaremba-Czogalla^4^, Marek Kubiszewski^5^, Marcin Cybulski^1,*^, Jerzy Gubernator^4^, Agnieszka Zagórska^6^ and Anna Jaromin^4,*^

1 Pharmacy, Cosmetic Chemistry and Biotechnology Research Group, Łukasiewicz Research Network-Industrial Chemistry Institute, Warsaw, Poland.

2 Dipartimento di Scienze Biomediche per la Salute, Università di Milano, Milan, Italy

3 Dipartimento di Scienze Biomediche, Chirurgiche e Odontoiatriche, Università di Milano, Milan, Italy

4 Department of Lipids and Liposomes, Faculty of Biotechnology, University of Wroclaw, Wroclaw, Poland

5 Pharmaceutical Analysis Laboratory, Łukasiewicz Research Network-Industrial Chemistry Institute, Warsaw, Poland

6 Department of Medicinal Chemistry, Jagiellonian University Medical College, Cracow, Poland

*Correspondence:

Anna Jaromin, Department of Lipids and Liposomes, Faculty of Biotechnology, University of Wroclaw, Joliot-Curie 14a, 50-383 Wroclaw, Poland

Email: [anna.jaromin@uwr.edu.pl](mailto:anna.jaromin@uwr.edu.pl)

Marcin Cybulski, Pharmacy, Cosmetic Chemistry and Biotechnology Research Group, Łukasiewicz Research Network-Industrial Chemistry Institute, Rydygiera 8, 01-793 Warsaw, Poland

Email: [marcin.cybulski@ichp.lukasiewicz.gov.pl](mailto:anna.jaromin@uwr.edu.pl)

List of ^1^H and ^13^C NMR spectra:

**Figure 1.** *tert*-Butyl (2*E*)-3-(2,4-dihydroxyphenyl)-propenoate (**1**)

**Figure 2.** Ethyl (2*E*)-3-(2,3,4-trihydroxyphenyl)-propenoate (**2**)

**Figure 3.** Ethyl (2*E*)-3-(2,3,4-trihydroxyphenyl)-2-methylpropenoate (**3**)

**Figure 4.***tert*-Butyl (2*E*)-3-(2,3,4-trihydroxyphenyl)- propenoate (**4**)

**Figure 5.**Ethyl (2*E*)-3-(3-fluoro-4-hydroxyphenyl)-2-methylpropenoate (**5**)

**Figure 6.** *tert*-Butyl (2*E*)-3-(3-fluoro-4-hydroxyphenyl)- propenoate (**6**)


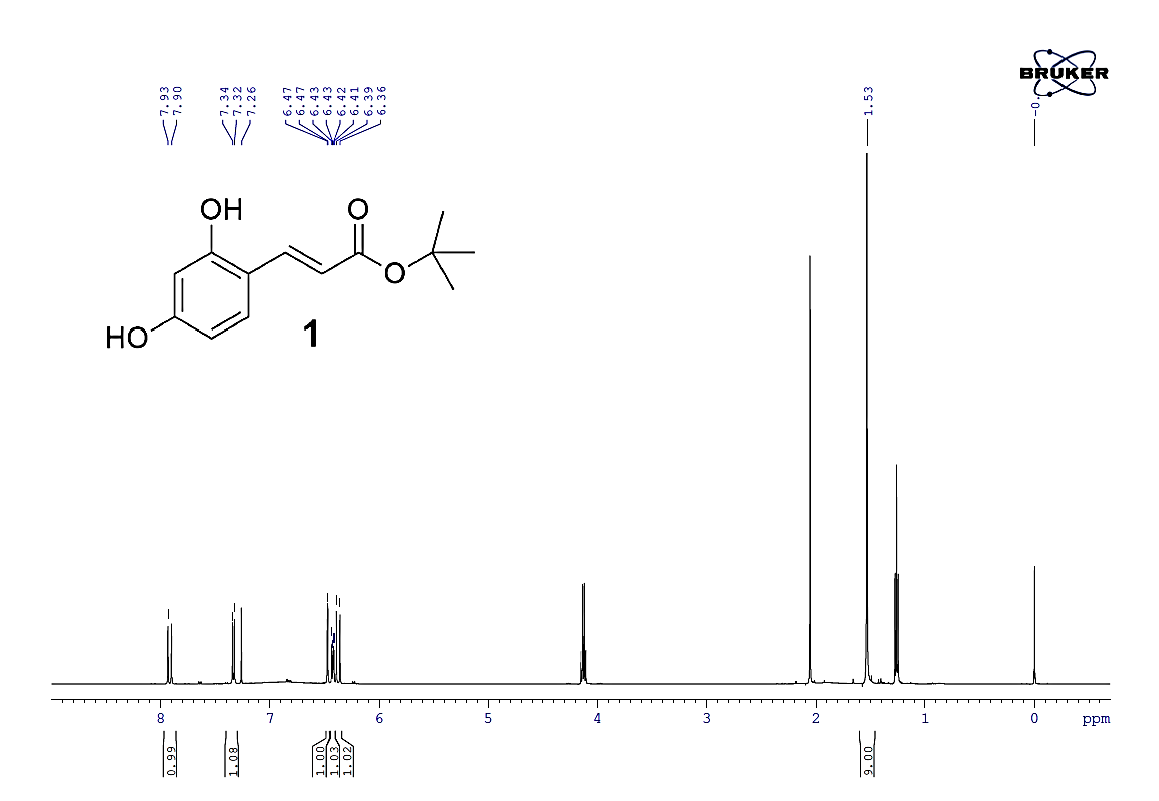


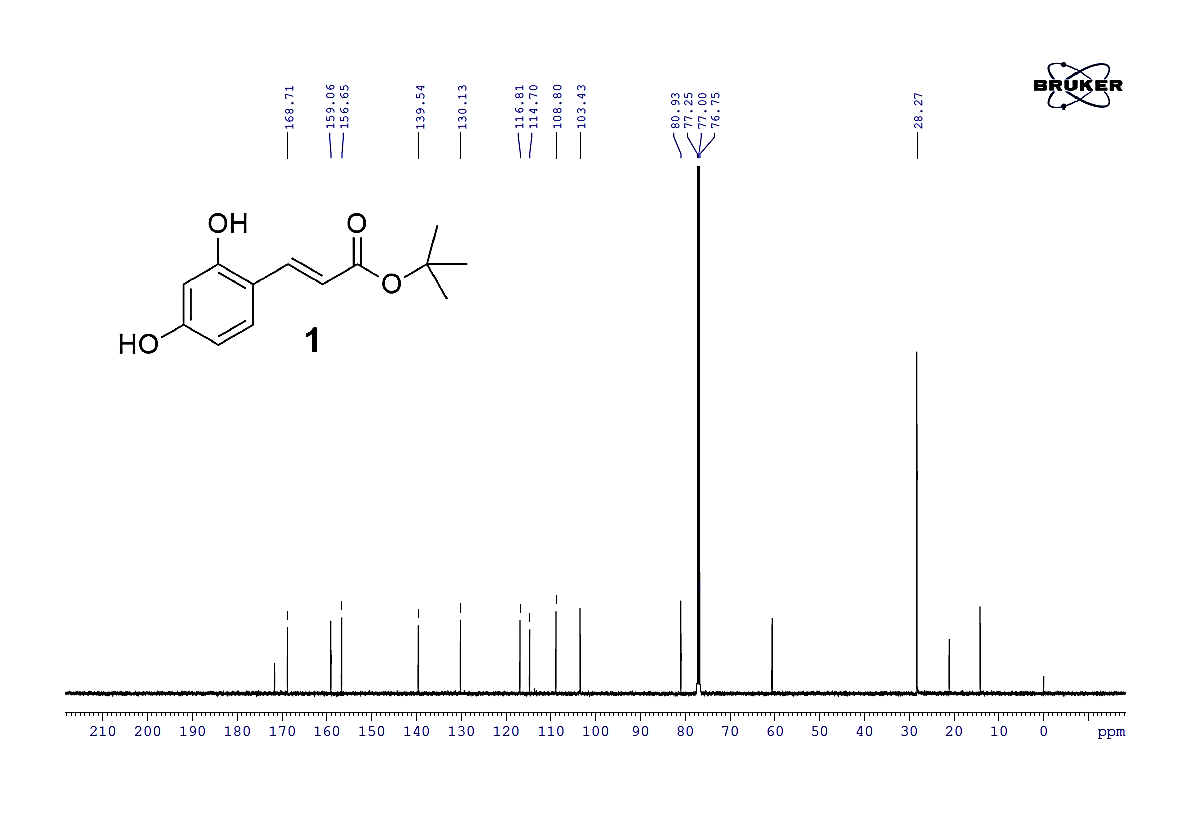


**Figure 1.** *tert*-Butyl (2*E*)-3-(2,4-dihydroxyphenyl)-propenoate (**1**)


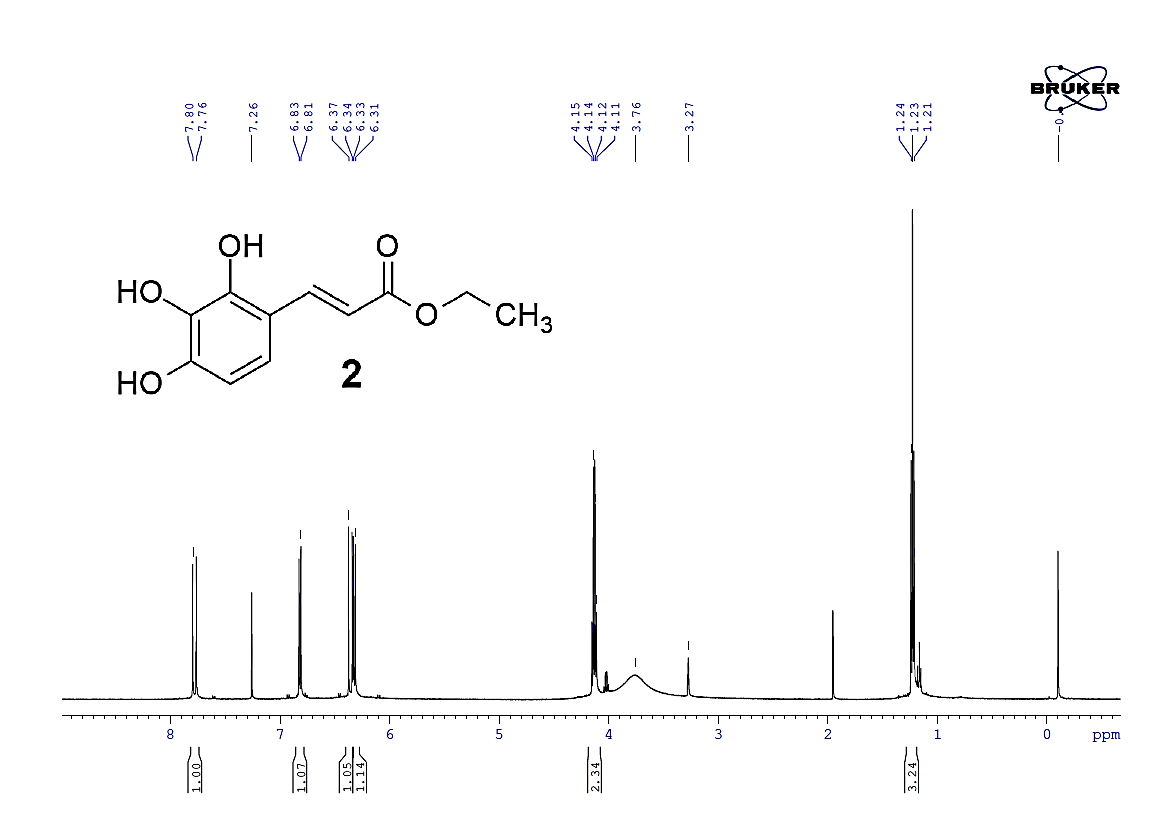


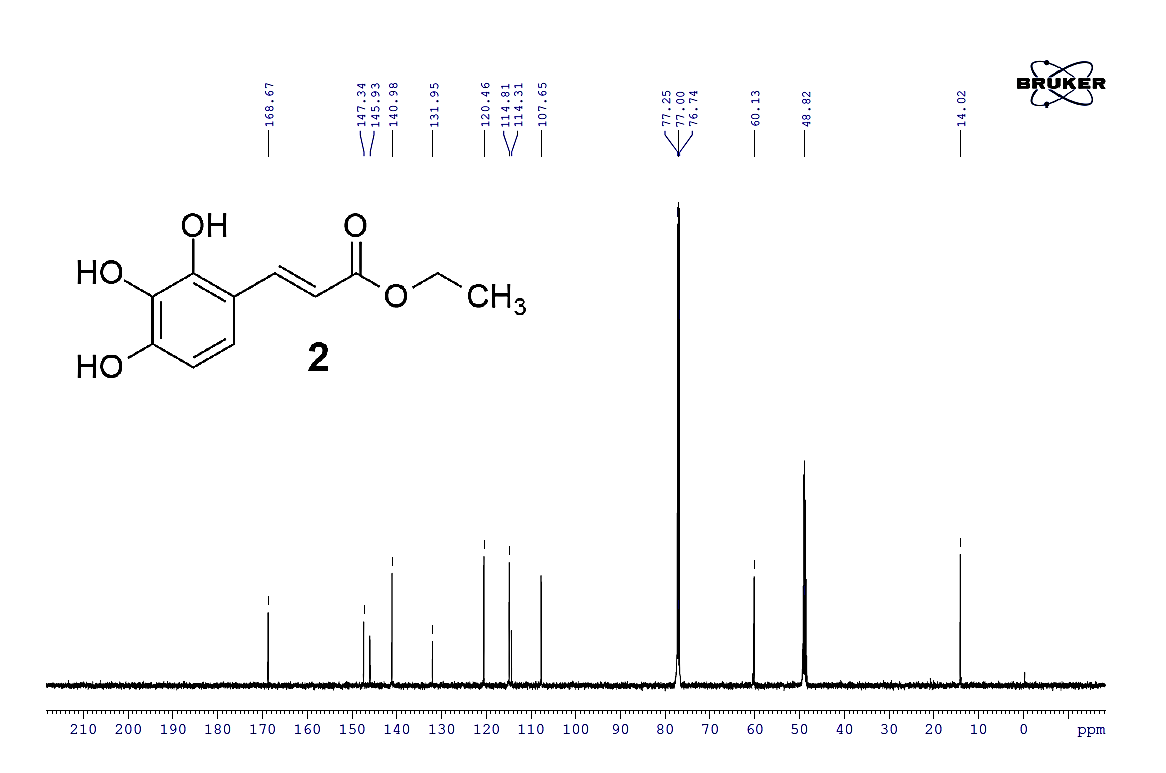


**Figure 2.** Ethyl (2*E*)-3-(2,3,4-trihydroxyphenyl)-propenoate (**2**)


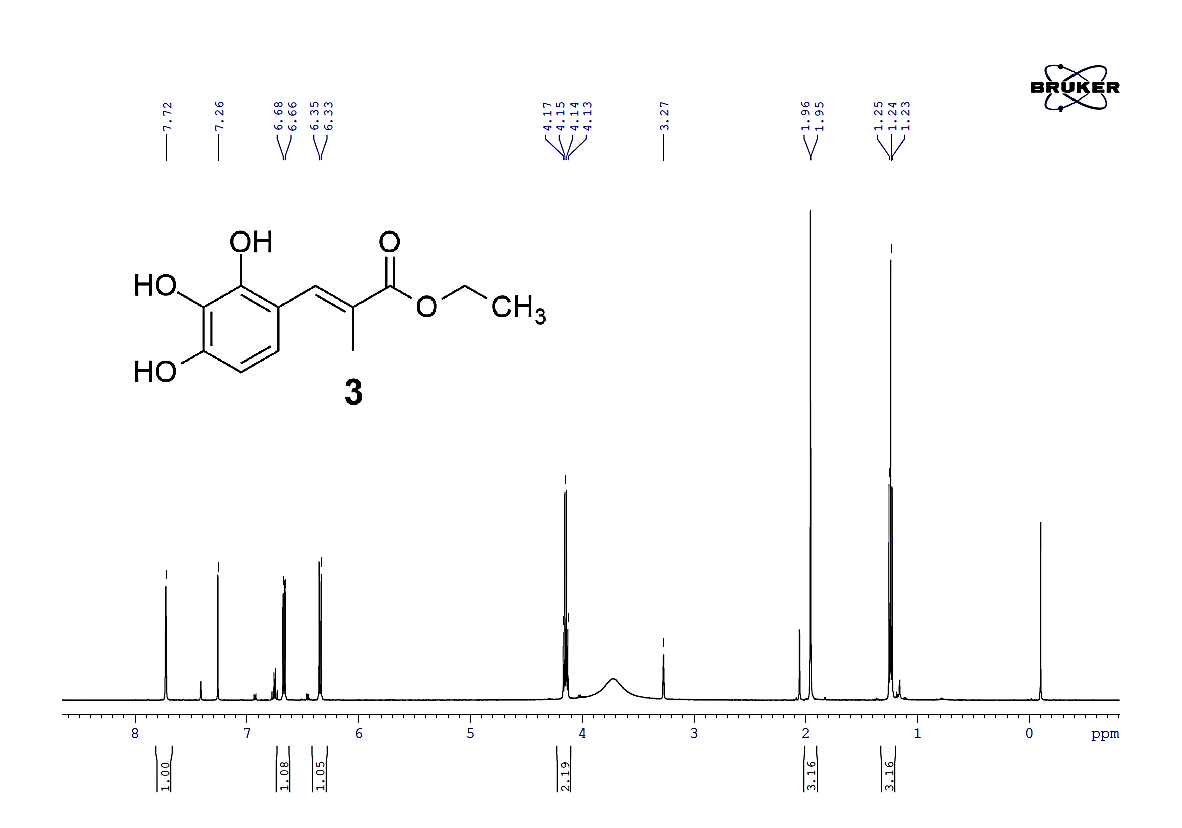


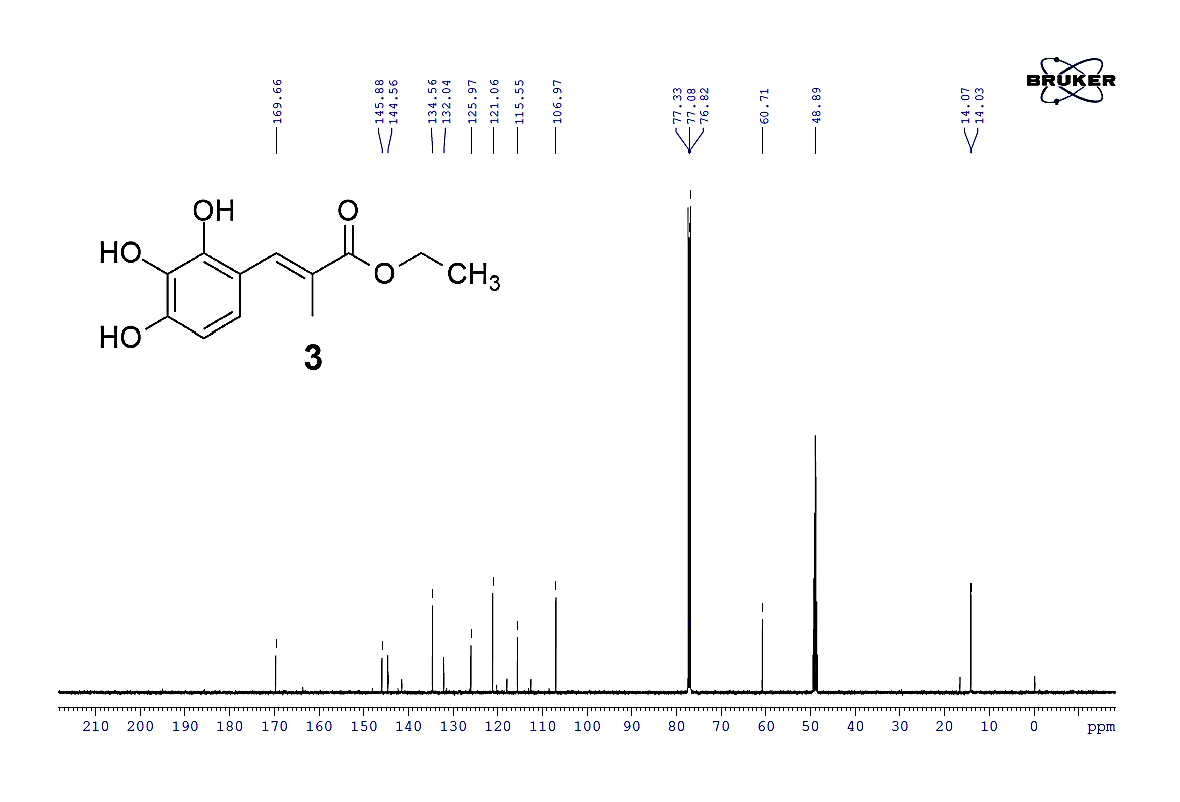


**Figure 3.** Ethyl (2*E*)-3-(2,3,4-trihydroxyphenyl)-2-methylpropenoate (**3**)


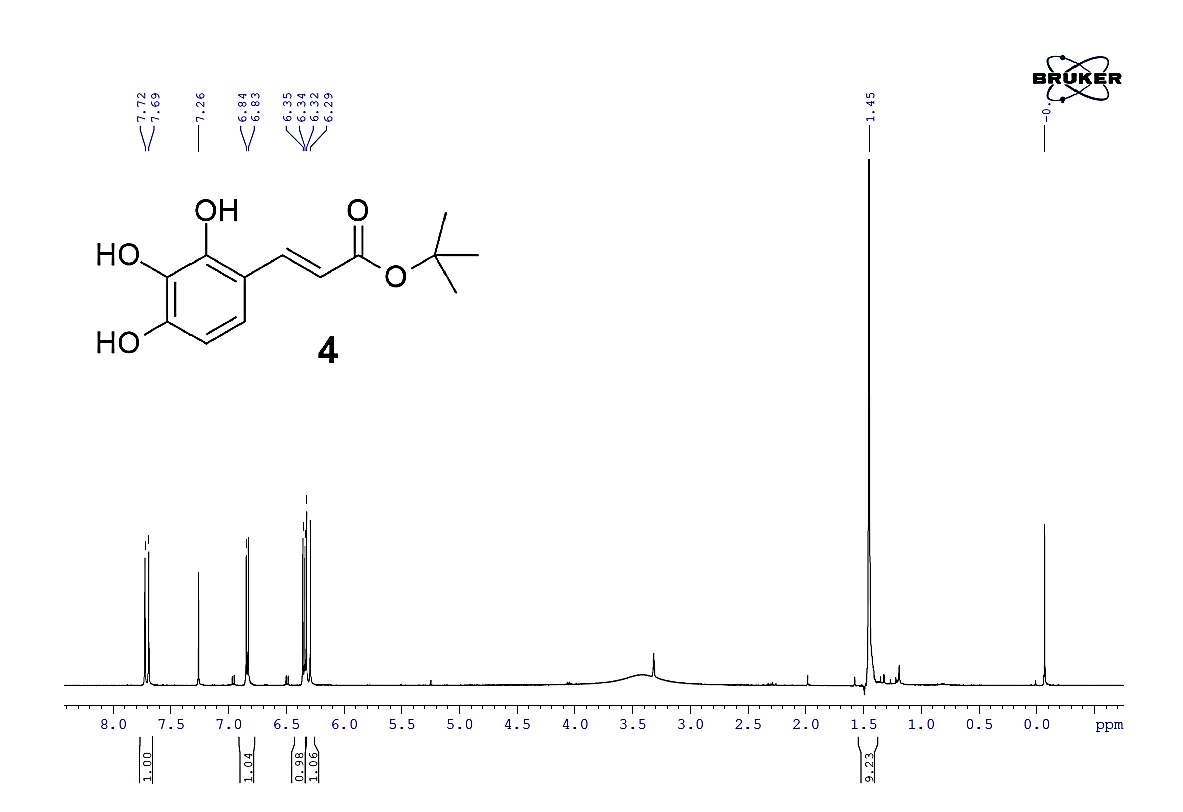


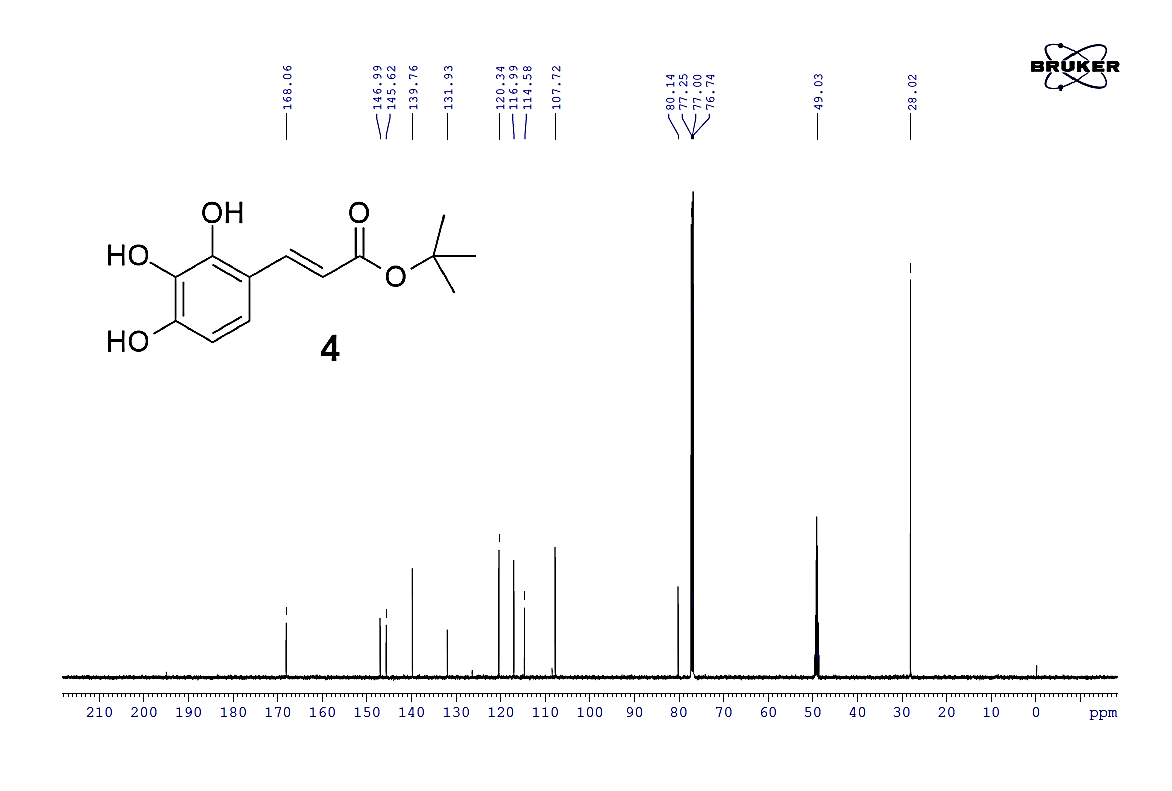


**Figure 4.***tert*-Butyl (2*E*)-3-(2,3,4-trihydroxyphenyl)- propenoate (**4**)


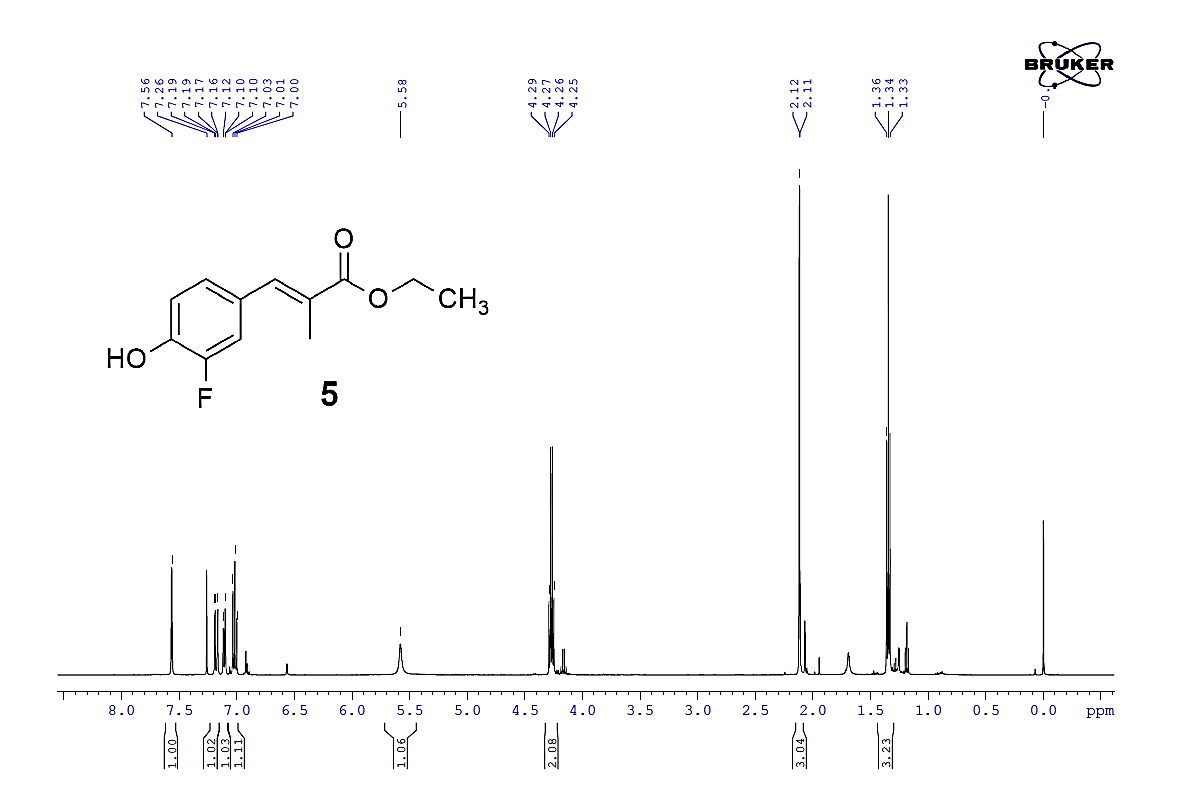


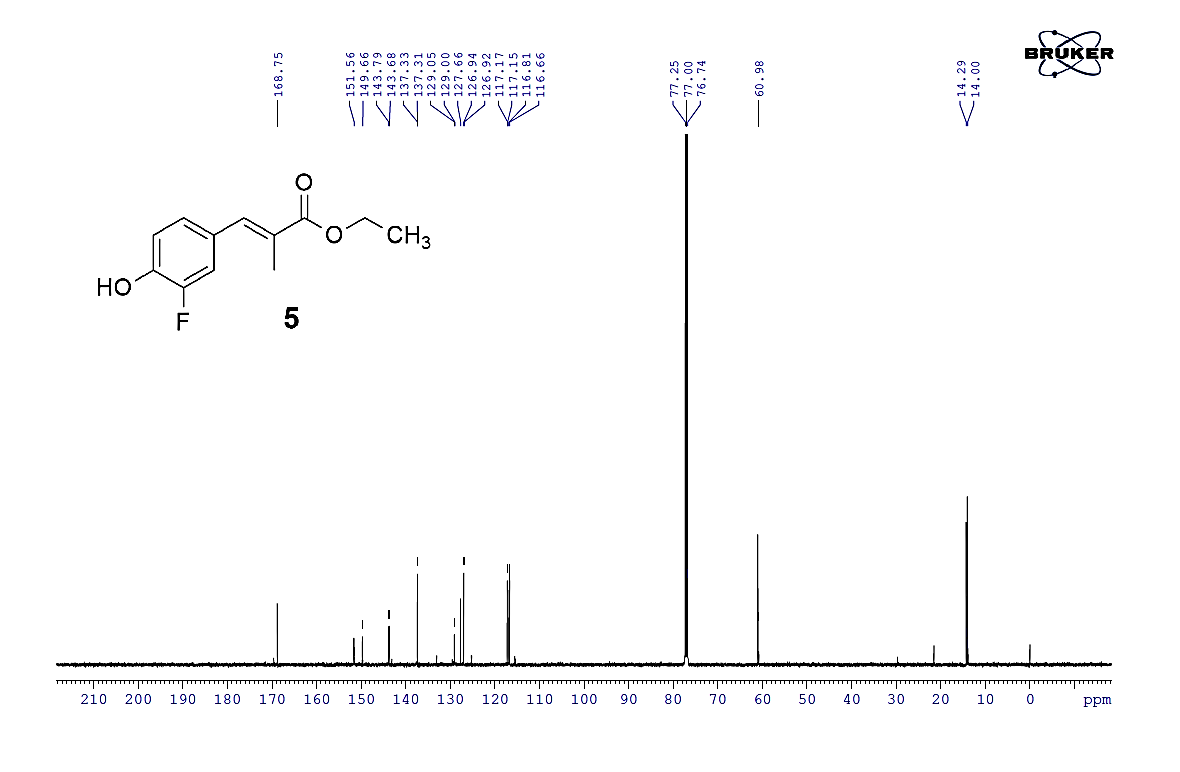


**Figure 5.**Ethyl (2*E*)-3-(3-fluoro-4-hydroxyphenyl)-2-methylpropenoate (**5**)


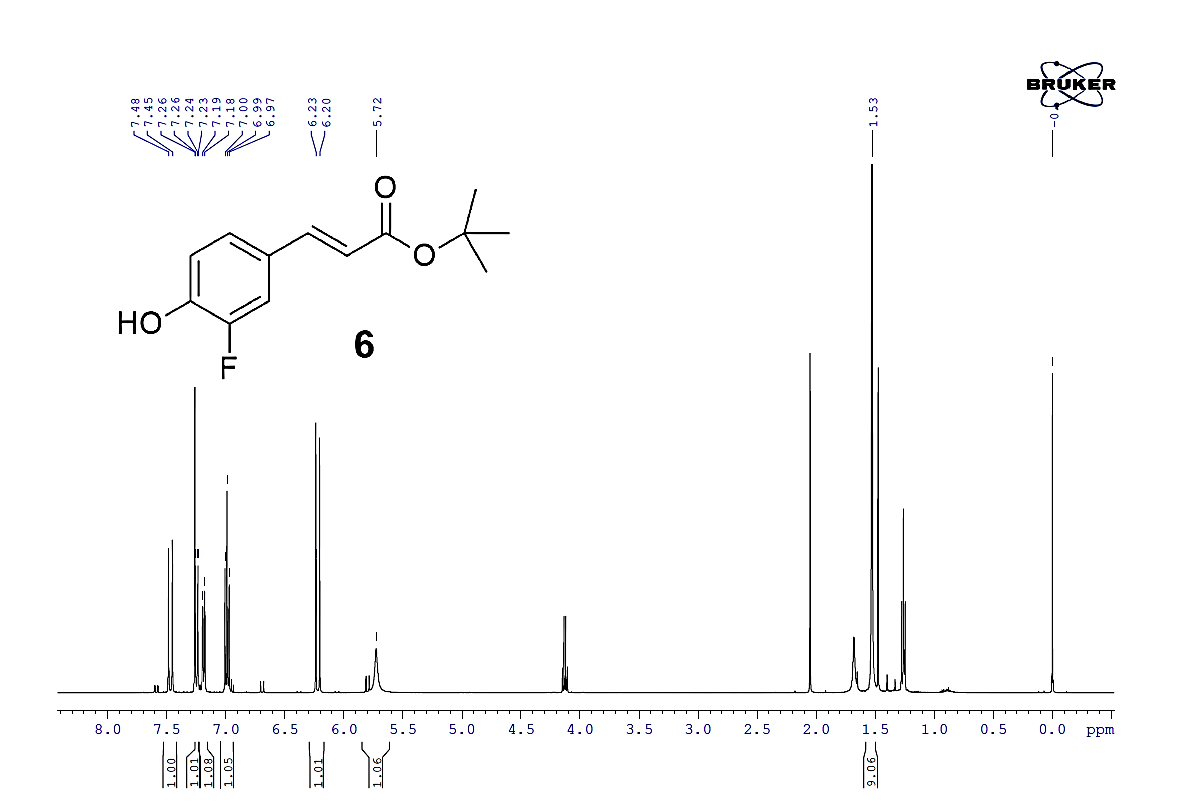


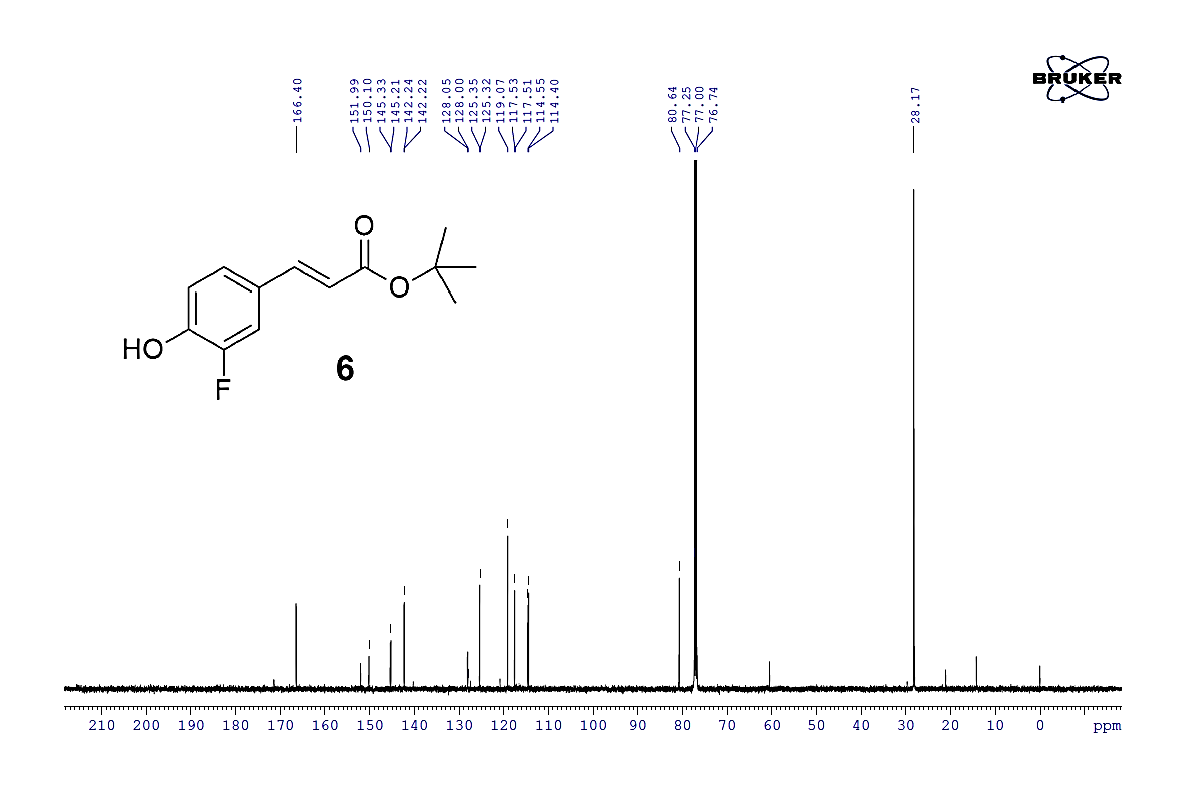


**Figure 6.** *tert*-Butyl (2*E*)-3-(3-fluoro-4-hydroxyphenyl)- propenoate (**6**)
